# Editorial: Current Insights Into LAMA2 Disease

**DOI:** 10.3389/fnmol.2021.780635

**Published:** 2021-11-18

**Authors:** Stefano C. Previtali, Ronald D. Cohn, Markus A. Ruegg

**Affiliations:** ^1^Institute of Experimental Neurology and Division of Neuroscience, Istituto di Ricerca e Cura a Carattere Scientifico (IRCCS) Ospedale San Raffaele, Milan, Italy; ^2^The Hospital for Sick Children, Toronto, ON, Canada; ^3^Department of Pediatrics and Molecular Genetics, University of Toronto, Toronto, ON, Canada; ^4^Biozentrum, University of Basel, Basel, Switzerland

**Keywords:** laminin, mouse model, clinical aspect, therapy, pathogenesis

Merosin Deficient Congenital Muscular Dystrophy type 1A (MDC1A; OMIM^*^156225), or LAMA2-Congenital Muscular Dystrophy (LAMA2-CMD) or LAMA2-Related Disease (LAMA2-RD), is an autosomal recessive disorder due to mutations in the *LAMA2* gene, which codes for the α2 chain of laminin-211. Laminin-211 (also called merosin) is an heterotrimer composed of α2, β1, and γ1 chain, and forming a key element in the formation of the basement membrane of skeletal muscle, peripheral nerve, and brain. As a consequence of *LAMA2* mutations, laminin-α2 containing heterotrimers are not assembled or are expressed at very low levels, causing progressive tissue degeneration. The disease has an estimated prevalence in UK and Italy of 0.6–0.7/100,000, and is characterized by a severe wasting muscular dystrophy, dysmyelinating neuropathy and brain abnormalities. Thus, it represents a multi-organ disorder with a preponderance of the muscle pathology and a wide range of severities from severe early-onset forms causing death in the first decade of life, to milder late-onset forms. Severe and milder forms are largely distinguished by the residual amount of laminin-211 expressed, and are both responsible of high social and economic costs due to chronic medications and hospitalizations in the absence of any effective therapy.

This Research Topic has generated a very informative collection of articles covering several aspects of LAMA2 disease, including molecular pathomechanisms, main clinical findings, lessons from animal models, development of potential treatments on the basis of mechanistic understanding, and the identification of potential biomarkers of the disease.

Sarkozy et al. introduce the clinical aspects of the disease, describing severe and milder forms, including typical hallmarks of early-onset forms, such as hypotonia, axial weakness, inability to achieve independent ambulation, and elevated creatine kinase (CK) levels in the blood. They also point out the presence of joints' contractures, feeding difficulties, respiratory dysfunction, central and peripheral nervous system involvement, and possible cardiac abnormalities. They finally provide indication for diagnosis and management of the disease, and discuss what is currently available on natural history studies and disease biomarkers to guide future clinical trials.

Previtali and Zambon dissert on the current knowledge of peripheral neuropathy in LAMA2 disease, summarizing all the findings reported in humans and animal models. They also present molecular mechansims responsible for LAMA2 neuropathy describing its pathological hallmark, the formation of bundles of unsorted axons, as a consequence of defective nerve development. Finally, they discuss the consequence of the disease on nerve regeneration, neuromuscular junctions, and potential therapeutic strategies.

Gawlik and Durbeej present a comprehensive overview of all the mouse models reproducing LAMA2 disease, describing their clinical aspects and lifespan, motor behavior, muscle pathology, and respiratory and heart function. They also show how clinical and pathological findings change with mouse age and discuss the molecular mechanisms sustaining the disease. Finally, they summarize pharmacological approaches to the disease in these mouse models.

Similar findings are reported by Fabian and Dowling in a different animal model, the zebrafish. They introduce this model as an excellent tool to investigate human muscle disorders in general, and present differences and similarities with the *LAMA2*-deficient zebrafish. Finally, results on drug screening and drug therapy in these animal models and potential caveats are discussed.

Arreguin and Colognato explore all the known findings on brain dysfunction in LAMA2 disease. They summarize data on laminin-211 expression in the central nervous system and its role in regulating the blood-brain-barrier, synaptic plasticity, axonal growth and pathfinding, neural stem cells and myelination. Moreover, they describe neurological, pathological, and imaging findings in human and mouse LAMA2 disease.

Accorsi et al. describe the central role of fibrosis and inflammation in the pathogenesis of LAMA2. The most significant driver of fibrosis is TGF-beta and its chronic dysregulation affects myofibroblast transdifferentiation and myogenesis. Finally, biomarkers of fibrosis and potential therapeutic strategies are discussed.

Yanay et al. dissert on the impairment of muscle regeneration in LAMA2 disease. They describe mechanisms of muscle regeneration and consequences of lack of laminin-211 impacting regeneration and repair. Finally, they present an overview of the signaling pathways involved and possible therapeutic strategies by interfering with these pathways to enhance regeneration.

The last two articles focus on specific therapeutic strategies to treat LAMA2 disease. In the article of Barraza-Flores et al., the authors discuss on the role of laminin-111 to ameliorate LAMA2 disease. They first present the effects of integrin-laminin interaction to regulate muscle structure and function, the role of laminin-211 and how laminin-111 can substitute it. They present data of the therapeutic effects of endogenous or exogenous laminin-111 protein on LAMA2 disease.

Finally, Yurchenco and McKee present findings of the use of linker proteins to repair LAMA2 basement membrane and thus improve LAMA2 disease. Following a detailed description of basement membrane composition and molecular players, they describe how mini-agrin and LNNd proteins can recruit laminin heterotrimers, other than laminin-221, to be polymerized and linked to laminin-211 receptors, in order to rebuild a proper and functional basement membrane in LAMA2 tissues and to ameliorate the disease.

We expect that the articles included in this Research Topic will expand knowledge and interest in the field of LAMA2 disease, in order conclude the characterization of the molecular mechanisms responsible of the disease, to develop extensive natural history studies and, finally, to achieve efficacious therapy(ies).

## Author Contributions

SP, RC, and MR edited the topic and wrote the editorial. All authors contributed to the article and approved the submitted version.

## Conflict of Interest

The authors declare that the research was conducted in the absence of any commercial or financial relationships that could be construed as a potential conflict of interest.

## Publisher's Note

All claims expressed in this article are solely those of the authors and do not necessarily represent those of their affiliated organizations, or those of the publisher, the editors and the reviewers. Any product that may be evaluated in this article, or claim that may be made by its manufacturer, is not guaranteed or endorsed by the publisher.

